# Effects of Tenascin C on the Integrity of Extracellular Matrix and Skin Aging

**DOI:** 10.3390/ijms21228693

**Published:** 2020-11-18

**Authors:** Young Eun Choi, Min Ji Song, Mari Hara, Kyoko Imanaka-Yoshida, Dong Hun Lee, Jin Ho Chung, Seung-Taek Lee

**Affiliations:** 1 Department of Biochemistry, College of Life Science and Biotechnology, Yonsei University, Seoul 03722, Korea; choii03271452@gmail.com; 2 Department of Dermatology, Seoul National University College of Medicine, Seoul 03080, Korea; minjisong@snu.ac.kr (M.J.S.); ivymed27@snu.ac.kr (D.H.L.); jhchung@snu.ac.kr (J.H.C.); 3 Department of Pathology and Matrix Biology, Mie University Graduate School of Medicine, Tsu 514-8507, Japan; marihara@doc.medic.mie-u.ac.jp (M.H.); imanaka@doc.medic.mie-u.ac.jp (K.I.-Y.); 4 Mie University Research Center for Matrix Biology, Tsu 514-8507, Japan; 5 Institute of Human-Environment Interface Biology, Medical Research Center, Seoul National University, Seoul 03080, Korea

**Keywords:** aging, collagen, extracellular matrix, fibroblast, skin, tenascin C, TGF-β

## Abstract

Tenascin C (TNC) is an element of the extracellular matrix (ECM) of various tissues, including the skin, and is involved in modulating ECM integrity and cell physiology. Although skin aging is apparently associated with changes in the ECM, little is known about the role of TNC in skin aging. In this study, we found that the *Tnc* mRNA level was significantly reduced in the skin tissues of aged mice compared with young mice, consistent with reduced TNC protein expression in aged human skin. TNC-large (TNC-L; 330-kDa) and -small (TNC-S; 240-kDa) polypeptides were observed in conditional media from primary dermal fibroblasts. Both recombinant TNC polypeptides, corresponding to TNC-L and TNC-S, increased the expression of type I collagen and reduced the expression of matrix metalloproteinase-1 in fibroblasts. Treatment of fibroblasts with a recombinant TNC polypeptide, corresponding to TNC-L, induced phosphorylation of SMAD2 and SMAD3. TNC increased the level of transforming growth factor-β1 (*TGF-β1*) mRNA and upregulated the expression of type I collagen by activating the TGF-β signaling pathway. In addition, TNC also promoted the expression of type I collagen in fibroblasts embedded in a three-dimensional collagen matrix. Our findings suggest that TNC contributes to the integrity of ECM in young skin and to prevention of skin aging.

## 1. Introduction

Tenascin C (TNC) is a multimodular extracellular matrix (ECM) protein with multiple molecular forms that are generated by alternative splicing and post-translational modifications [1]. TNC contains an assembly domain essential for the formation of hexamers, epidermal growth factor (EGF)-like repeats, fibronectin type III-like repeats, and a C-terminal fibrinogen-like globular domain.

TNC is mainly expressed in distinct spatial and temporal patterns during embryonic development. TNC is weakly expressed in quiescent adult tissues but is strongly expressed under various pathological conditions such as tissue injury, wound healing, inflammation, and tumorigenesis [2,3,4,5,6].

Although TNC is not an obligatory structural element of the ECM, it binds to structural proteins and cell surface receptors such as the EGF receptor and integrins in the ECM [7,8,9,10]. Binding of TNC to these receptors activates their downstream pathways and affects cell proliferation, adhesion, and migration, depending on cell types and environments [11,12,13,14,15].

TNC is known to upregulate the expression of type I collagen in foreskin fibroblasts and hepatic stellate cells [16,17]. The expression of TNC is elevated in collagen diseases [16] that are characterized by inflammation, autoimmune attack, and vascular damage and often leads to fibrosis [18]. Patients with increased TNC levels show a higher incidence of diffuse cutaneous systemic sclerosis, severe thickened skin, and probability of pulmonary fibrosis compared to those with normal levels.

Transforming growth factor-β (TGF-β) upregulates collagens and downregulates matrix metalloproteinases (MMPs), the major enzymes involved in degrading collagens and ECM components, and also contributes to the prevention of collagen loss in aged human skin [19]. TNC deficiency attenuates TGF-β-mediated fibrosis following immune-mediated chronic hepatitis [20] or acute lung injury [21]. TNC induces activation of hematopoietic stem cells mediated by TGF-β1 and α9β1 integrin, thereby elevating type 1 collagen production and promoting cell migration [17]. TNC also activates the TGF-β signaling pathway and induces the expression of type I collagen, at least in fibrotic lesions.

Previous studies suggest that TNC is implicated in collagen biosynthesis and is relevant to TGF-β signaling in fibroblasts. However, the role of TNC in normal skin, particularly during aging, has not been studied. In the present study, we investigated the expression of TNC in skin tissues from young and aged mice and humans by reverse transcription-polymerase chain reaction (RT-PCR) and histological analysis. The major forms of TNC that are expressed in human primary dermal fibroblasts were also analyzed. Next, the major forms of human TNC were ectopically expressed, and the effect of recombinant TNC polypeptides on the secretion of type I collagen and MMP-1 in foreskin fibroblasts was analyzed. We then analyzed the molecular mechanism of TNC-induced upregulation of type I collagen. In addition, the effect of TNC on the synthesis of type I collagen was validated in fibroblasts cultured in a three-dimensional (3D) collagen matrix. Based on our findings, we suggest that TNC is an important molecule that maintains the ECM integrity and prevents and attenuates skin aging.

## 2. Results

### 2.1. Expression of TNC is Downregulated during Intrinsic Skin Aging in Mouse and Human Skin Tissues

**Figure 1:**
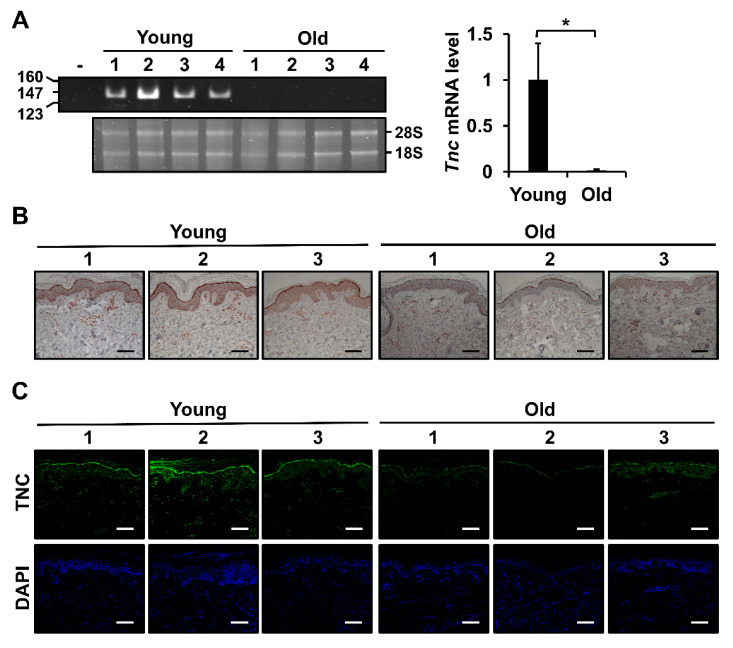
Analysis of tenascin C (TNC) expression in skin tissues from young and old mice and humans. (**A**) *Tnc* mRNA level was analyzed in mouse skin tissues by reverse transcription-polymerase chain reaction (RT-PCR). The panel on the right shows a graphical presentation of *Tnc* mRNA levels in mouse skin tissues quantified using multi-gauge densitometry software. *Tnc* mRNA level relative to that in skin tissues of young mice (n = 4) is shown as mean ± SD. * *p* < 0.05. -: no template as a negative control. (**B**,**C**) Immunohistochemical (IHC) (**B**) and immunofluorescence (IF) (**C**) analyses of TNC protein level in skin tissues from young and elderly human subjects. Sections of skin tissues were incubated with TNC antibody. For IHC, the specimens were stained with horseradish peroxidase-conjugated secondary antibody and 3-amino-9-ethylcarbazole and counterstained with hematoxylin. For IF, the specimens were incubated with secondary antibody conjugated with Alexa Flour 488 (green) and counterstained with 4′,6-diamidino-2-phenylindole (DAPI) (blue). Magnification, × 200. Bar = 100 µm.

To analyze the changes in *Tnc* mRNA level in mouse skin tissues during aging, *Tnc* mRNA levels in the skin tissues of young (3 months old) and aged (24 months old) mice were determined by RT-PCR. *Tnc* mRNA levels showed a significant decrease in the skin tissues of aged mice compared with young mice (Figure 1A). To examine the aging-associated changes in TNC level in human skin, immunohistochemical (IHC) and immunofluorescence (IF) analyses were performed in the sun-protected buttock skin tissues of young (in their 30s) and elderly (in their 70s) females. IHC staining of young skin tissues revealed that TNC signals were present in the dermis as well as the epidermis, particularly in the basal layer (Figure 1B). However, TNC staining was barely detectable in aged skin tissues (Figure 1B). The pattern of TNC staining by IF analysis was consistent with that by IHC analysis. In addition, more TNC-positive cells and higher TNC signals were evident in the dermis of young skin tissues than in the dermis of elderly skin tissues (Figure 1C).

### 2.2. TNC Upregulates Type I Collagen Expression and Downregulates MMP-1 Expression in Fibroblasts

**Figure 2:**
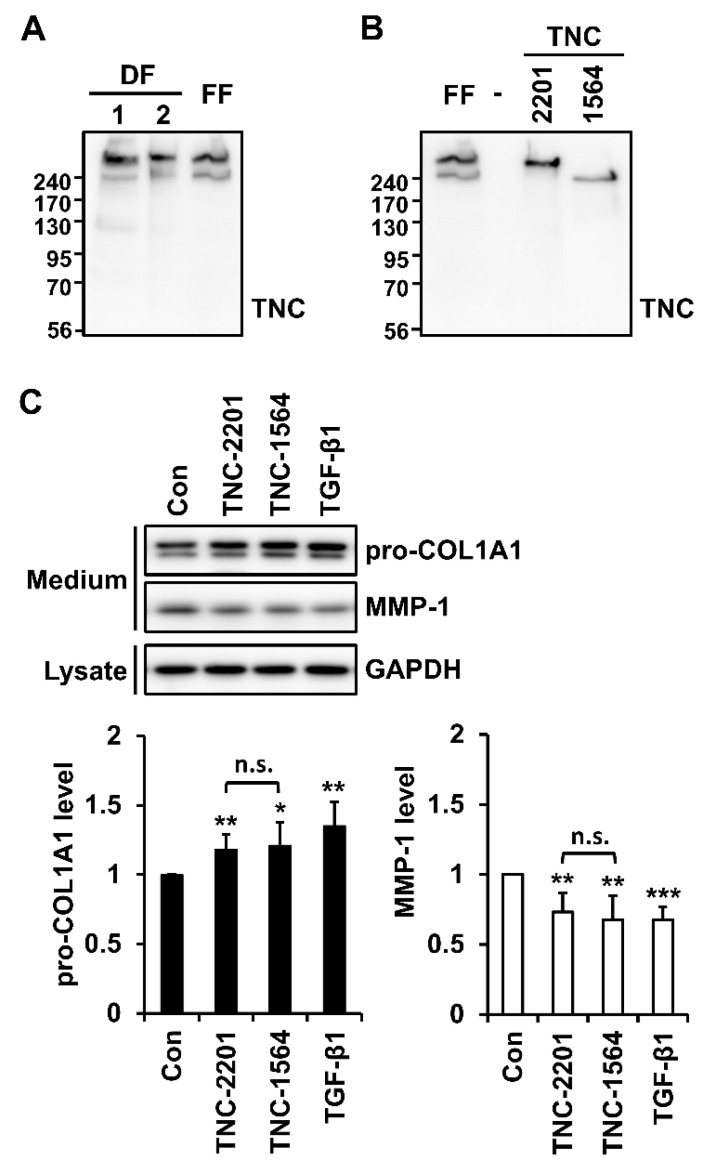
Detection of expression of TNC isoforms in fibroblasts, ectopic expression of recombinant TNC isoforms, and their effects on the secretion of type I collagen and matrix metalloproteinase-1 (MMP-1) in fibroblasts. (**A**) TNC levels were analyzed in conditioned media from two independent lines of human dermal fibroblasts (DF) and one foreskin fibroblast (FF) line by western blotting with an antibody against TNC. (**B**) Constructs expressing the large (TNC-2201) and small (TNC-1564) TNC isoforms were transfected into COS-1 cells, and the purified TNC proteins were analyzed by western blotting with TNC antibody. -: vector control. (**C**) Human foreskin fibroblasts were incubated with serum-free Dulbecco’s modified Eagle’s medium (DMEM) in the absence (Con) or presence of TNC (2 µg/mL) or transforming growth factor-β1 (TGF-β1) (3 ng/mL) for 24 h. Levels of type I collagen and MMP-1 were evaluated in conditioned media and that of GAPDH in cell lysates by western blot analysis using antibodies against pro-COL1A1, MMP-1, and GAPDH. Graphs show the relative protein levels of pro-COL1A1 and MMP-1. Each value represents the mean ± SD of six independent experiments. * *p* < 0.05, ** *p* < 0.01, *** *p* < 0.001 vs. Con. n.s.: not significant.

The size of the TNC monomers varies due to alternative splicing [1]. When TNC polypeptides secreted by two independent lines of human dermal fibroblasts and one foreskin fibroblast line were analyzed by western blotting, two major TNC isoforms (L and S) were detected. The expression of the larger isoform of TNC (TNC-L) was higher than that of the smaller isoform (TNC-S) in the human fibroblasts (Figure 2A).

It has been reported that TNC isoforms secreted from dermal fibroblasts are similar in apparent molecular weights (MWs) to TNC isoforms consisting of 2201 amino acid residues (TNC-2201) and 1564 residues (TNC-1564) [22,23]. Thus, we directly compared the size of TNC isoforms secreted from fibroblasts with recombinant TNC-2201 and TNC-1564 isoforms ectopically expressed in COS-1 cells. We found that the apparent MWs of TNC-L and TNC-S expressed in fibroblasts are identical to those of recombinant TNC-2201 and TNC-1564, respectively (Figure 2B).

We next analyzed whether TNC could induce changes in the expression of type I collagen and MMP-1 in foreskin fibroblasts. Cells were treated with recombinant human TNC-2201 and TNC-1564 polypeptides as well as TGF-β1 (as a positive control). Both recombinant TNC polypeptides as well as TGF-β1 caused a significant increase in type I collagen and a significant decrease in MMP-1 at the protein level (Figure 2C). The two TNC isoforms, however, showed no difference in the increase of type I collagen level and the decrease of MMP-1 level (Figure 2C). Therefore, a recombinant TNC-2201 polypeptide was used for further analysis of TNC.

**Figure 3:**
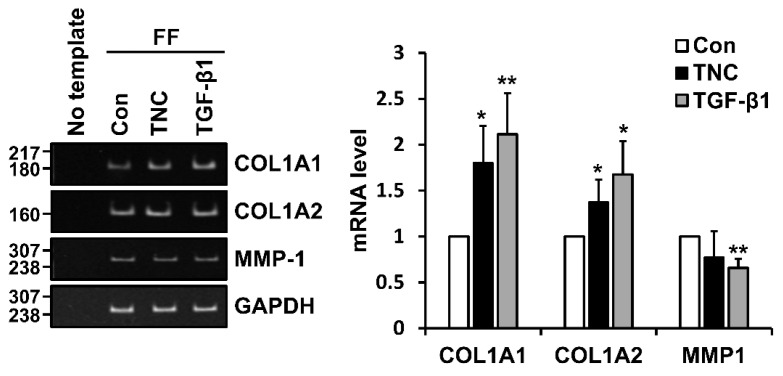
Effect of TNC on the levels of type I collagen and *MMP-1* mRNAs in human fibroblasts. Serum-starved human foreskin fibroblasts (FF) were incubated without (Con) or with TNC (2 µg/mL) or TGF-β1 (3 ng/mL) for 12 h. Levels of *COL1A1*, *COL1A2*, and *MMP-1* mRNAs were evaluated by conventional (left) and quantitative (right) RT-PCR analyses. Each value represents the mean ± SD of five independent experiments. * *p* < 0.05, ** *p* < 0.01 vs. Con.

The mRNA levels of *COL1A1*, *COL1A2*, and *MMP-1* with or without TNC treatment were analyzed in foreskin fibroblasts by conventional and quantitative RT-PCR. As expected, *COL1A1* and *COL1A2* mRNA levels were significantly upregulated following treatment with TNC as well as TGF-β1 (Figure 3). However, *MMP-1* mRNA level was decreased following TNC treatment, although the difference was not statistically significant (Figure 3).

### 2.3. TNC Activates Receptor-Regulated SMADs (R-SMADs) and TGF-β Receptors in Fibroblasts

**Figure 4:**
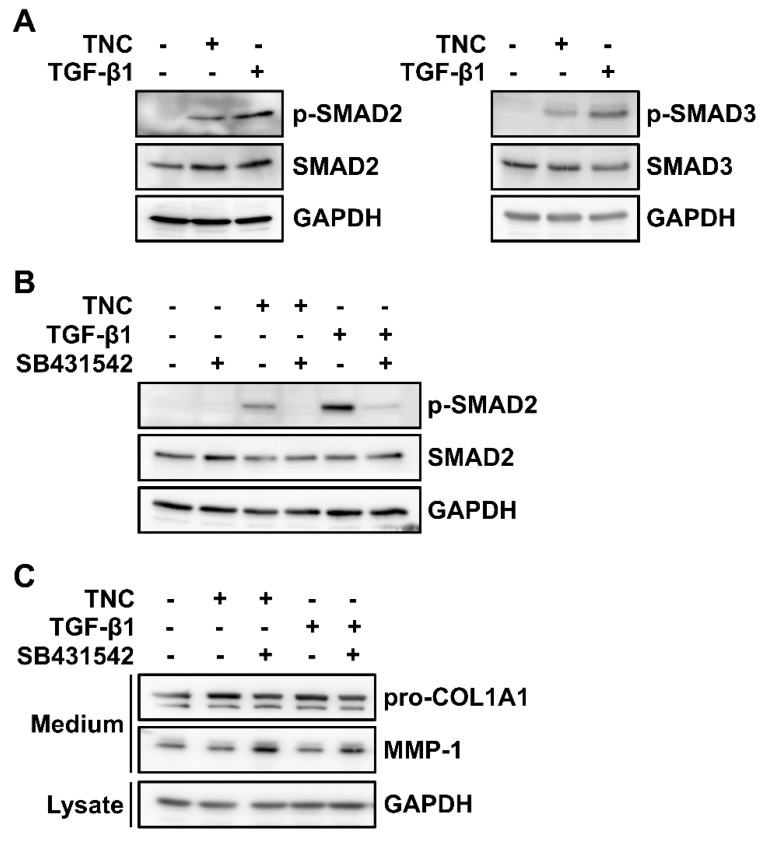
Analysis of receptor-regulated SMAD activation by TNC in fibroblasts. (**A**) Subconfluent human foreskin fibroblasts were starved for 12 h and then incubated with TNC (2 µg/mL) or TGF-β1 (3 ng/mL) for 90 min. Cell lysates were analyzed by western blotting using antibodies against phospho-SMAD2 (p-SMAD2), SMAD2, phospho-SMAD3 (p-SMAD3), SMAD3, and GAPDH. (**B**) Serum-starved foreskin fibroblasts were preincubated with SB431542 (10 µM) for 10 min and then stimulated with TNC (2 µg/mL) or TGF-β1 (3 ng/mL) for 90 min. Cell lysates were analyzed by western blotting with antibodies against p-SMAD2, SMAD2, and GAPDH. (**C**) Serum-starved foreskin fibroblasts were stimulated with TNC (2 µg/mL) or TGF-β1 (3 ng/mL) in the presence of SB431542 (10 µM) for 24 h. Levels of type I collagen and MMP-1 in conditioned media and GAPDH in cell lysates were evaluated by western blot analysis using antibodies against pro-COL1A1, MMP-1, and GAPDH.

To elucidate whether the TNC-mediated induction of type I collagen expression involves TGF-β signaling pathway, we analyzed the activation of representative R-SMADs, SMAD2, and SMAD3 in foreskin fibroblasts following TNC treatment. Treatment with TNC (2 µg/mL) as well as TGF-β1 (3 ng/mL) increased the phosphorylation of SMAD2 and SMAD3 (Figure 4A). Next, the effect of SB431542, an inhibitor of TGF-β receptor type I, was examined on TNC-induced SMAD2 activation in foreskin fibroblasts. Treatment with SB431542 abolished SMAD2 phosphorylation induced by TNC and severely impaired TGF-β1-induced phosphorylation (Figure 4B). In addition, SB431542 inhibited TNC-induced type I collagen secretion, whereas it restored TNC-mediated suppression of MMP-1 secretion (Figure 4C). These results demonstrate that TNC induces type I collagen expression via activation of TGF-β receptors and R-SMADs.

**Figure 5:**
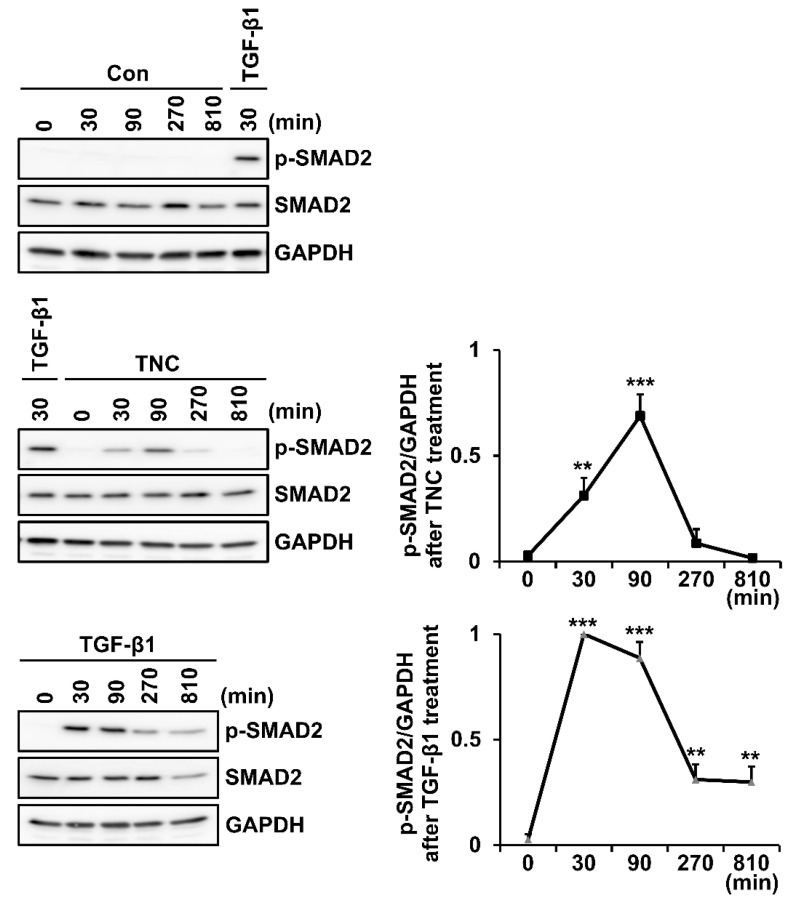
Time course effect of TNC on SMAD2 phosphorylation in fibroblasts. Serum-starved human foreskin fibroblasts were treated with vehicle (Con), TNC (2 µg/mL), or TGF-β1 (3 ng/mL). After incubation for the indicated durations (0, 30, 90, 270, and 810 min), cell lysates were analyzed by western blotting using antibodies against phospho-SMAD2 (p-SAMD2), SMAD2, and GAPDH. Relative intensity of p-SMAD2/GAPDH in samples treated with TNC or TGF-β1, normalized to a sample treated with TGF-β1 for 30 min, are shown in graphs. Each value represents the mean ± SD of four independent experiments. ** *p* < 0.01, *** *p* < 0.001 vs. 0 min.

We further performed the time-course analysis of SMAD2 phosphorylation following treatment with TNC or TGF-β1 for up to 810 min in foreskin fibroblasts. Phosphorylation of SMAD2 peaked 90 min after TNC treatment, but it peaked 30 min after TGF-β1 treatment (Figure 5). Based on these results, we speculate that TNC activates TGF-β receptors in a different way than TGF-β1.

### 2.4. TNC Induces TGF-β Family Members in Fibroblasts

**Figure 6:**
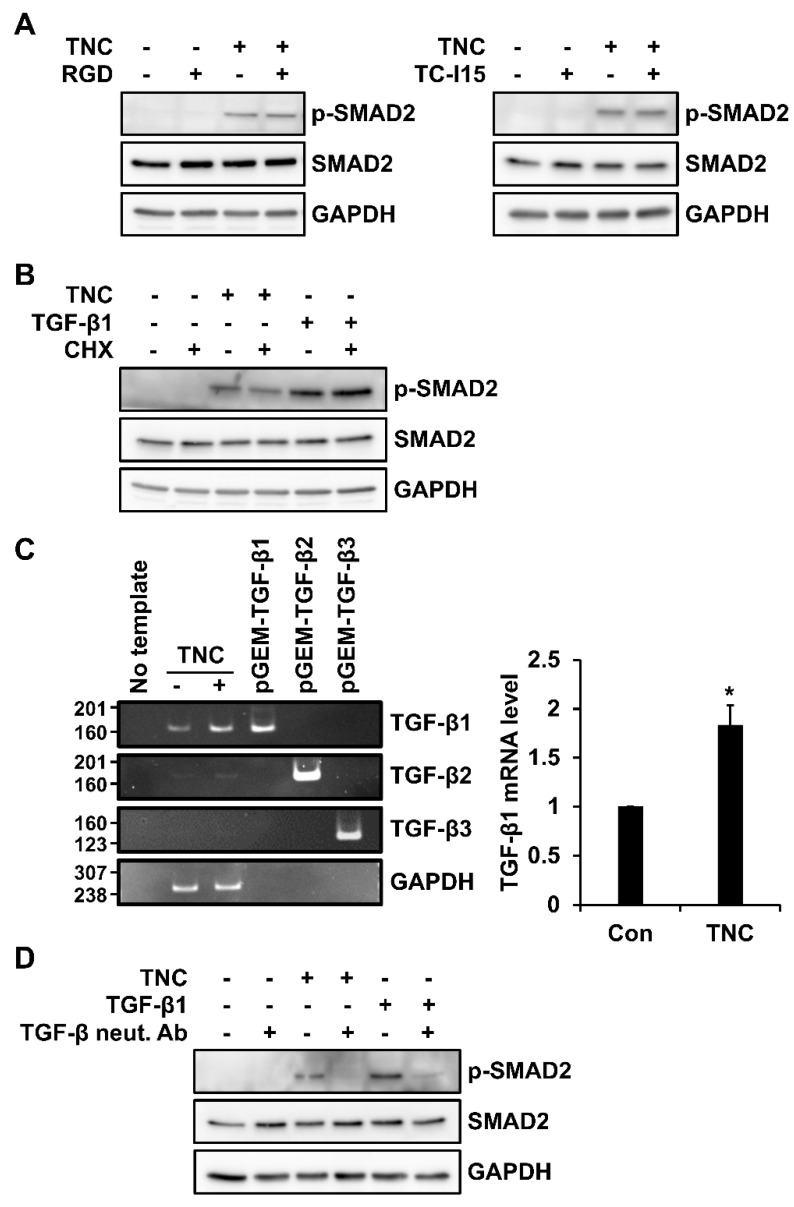
Analysis of the involvement of TGF-β1 in TNC-induced SMAD2 activation in fibroblasts. (**A**,**B**) Subconfluent human foreskin fibroblasts were starved for 12 h and then incubated with RGD peptide (100 µg/mL) or TC-I15 (2 µM) (**A**) or cycloheximide (CHX; 10 µg/mL) (**B**) for 10 min and then stimulated with TNC (2 µg/mL) or TGF-β1 (3 ng/mL) for 90 min. Cell lysates were analyzed by western blotting with antibodies against p-SMAD2, SMAD2, and GAPDH. (**C**) Subconfluent human foreskin fibroblasts were incubated in the absence or presence of TNC (2 µg/mL) for 12 h. *TGF-β1*, *TGF- β2*, and *TGF-β3* mRNA levels were evaluated by conventional RT-PCR analysis using 1 ng of pGEM-TGF-β1, 2, and 3 as controls and further by quantitative RT-PCR analysis. Graph on the right shows the relative level of *TGF-β1* mRNA normalized to *GAPDH* mRNA level. Each value represents the mean ± SD of three independent experiments. * *p* < 0.05 vs. Con. (**D**) Subconfluent human foreskin fibroblasts were incubated with TGF-β neutralizing antibody (TGF-β neut. Ab; 3 µg/mL) and analyzed for western blotting, as described in (**A**,**B**).

Integrins are known to induce TGF-β expression or activate latent forms of TGF-β [24]. To determine whether integrins are involved in TNC-mediated SMAD activation, the activation of integrins was blocked by treatment with RGD peptide or TC-I15. However, RGD peptide and TC-I15 did not affect TNC-induced SMAD2 activation in foreskin fibroblasts (Figure 6A).

To investigate whether TNC induces the biosynthesis of TGF-β members, foreskin fibroblasts were treated with cycloheximide, a translation inhibitor, along with TNC or TGF-β1. While cycloheximide did not decrease TGF-β1-induced SMAD2 phosphorylation, it significantly reduced TNC-induced SMAD2 phosphorylation (Figure 6B), suggesting that TNC-induced activation of TGF-β receptors involves the biosynthesis of a signaling molecule.

We next analyzed the TNC-induced modulation of expression of TGF-β family members in foreskin fibroblasts. Conventional and quantitative RT-PCR analyses showed that TNC increased the level of *TGF-β1* mRNA (Figure 6C). Although the mRNA levels of *TGF-β2* and *TGF-β3* were very low, TNC also increased their mRNA levels (Figure 6C). In addition, treatment with a TGF-β neutralizing antibody (3 µg/mL) inhibited both TNC-induced SMAD2 phosphorylation (Figure 6D). These results demonstrate that TNC-induced SMAD2 activation involves the induction of TGF-β1.

### 2.5. TNC Increases the Biosynthesis of Type I Collagen during 3D Culture of Fibroblasts

To examine whether TNC affects ECM integrity in young mice, the skin tissues of young (6 weeks old) *Tnc* knockout (KO) and wild-type (WT) mice (n = 5) were stained with Masson’s trichrome, and the dermal thickness and collagen density in the dermis were measured. Both dermal thickness and collagen intensity were reduced in skin tissues from *Tnc* KO mice compared with WT mice, albeit not statistically significant (Supplementary Figure S1).

**Figure 7:**
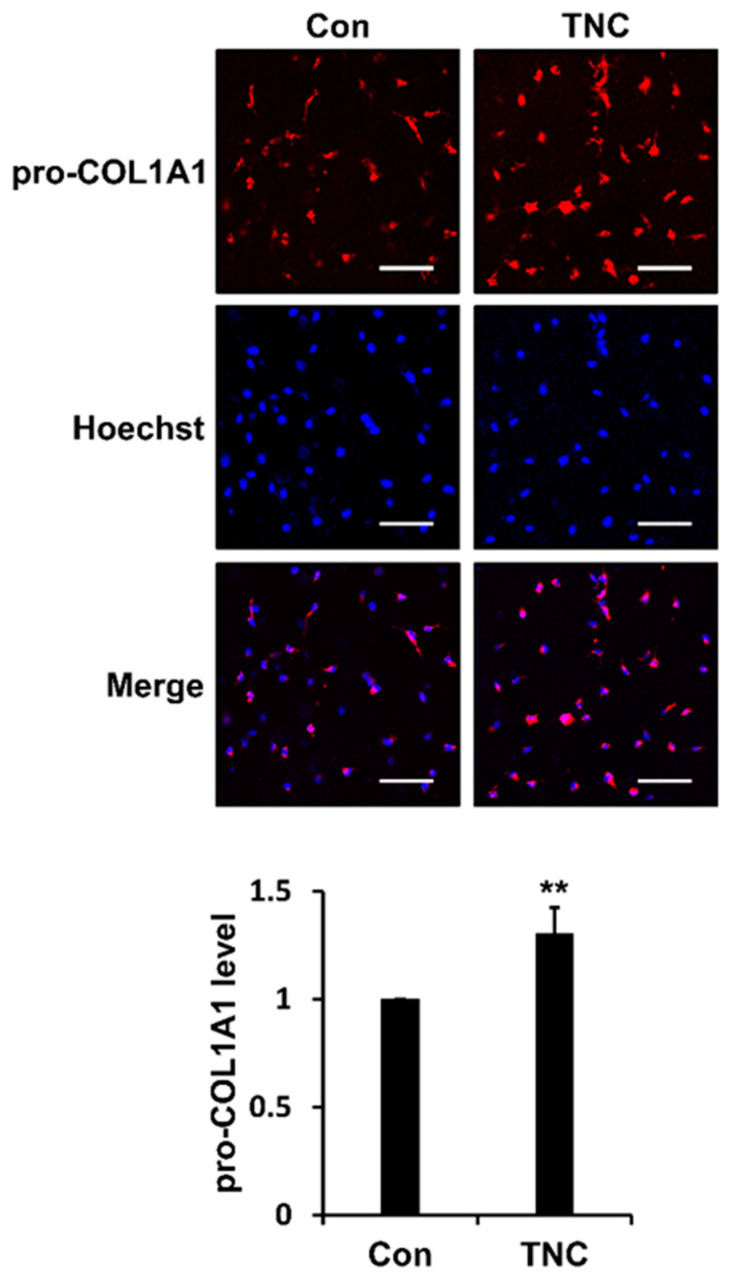
Effect of TNC on secretion of type I collagen during three-dimensional (3D) culture of fibroblasts. Foreskin fibroblasts embedded within a 3D type I collagen matrix without (Con) or with TNC (2 µg/mL) were incubated in serum-free DMEM for 24 h. The 3D matrix containing fibroblasts was stained with pro-COL1A1 primary antibody and Rhodamine Red-X secondary antibody, and with Hoechst 33258 for nuclear staining. Cells were analyzed by confocal fluorescence microscopy (×200). Relative pro-COL1A1 staining intensity normalized to nuclear staining intensity is shown in a graph. Each value represents the mean ± SD of three independent experiments. ** *p* < 0.01 vs. Con. Bar = 100 µm.

To analyze the effect of TNC on ECM integrity under in vivo-mimicking conditions, we investigated whether TNC affects the production of type I collagen in the 3D cultures of foreskin fibroblasts. Interestingly, TNC significantly increased the amount of newly synthesized type I collagen under 3D culture condition (Figure 7). These data suggest that TNC plays an important role in maintaining the ECM integrity of connective tissues such as skin.

## 3. Discussion

TNC is expressed at relatively low levels in normal adult human skin. However, TNC expression is significantly elevated in the dermal compartment during wound healing and in skin tumors [25,26]. We found that the level of *Tnc* mRNA was downregulated in the dorsal skin tissue of aged mice compared with young mice. In addition, IHC staining showed that TNC levels in sun-protected skin tissues of elderly females were markedly lower than those of young females. Moreover, the expression of type I procollagen mRNA and protein in sun-protected skin tissues gradually decreased during the intrinsic aging process [27]. Based on these results, we hypothesize that TNC is an important molecule involved in intrinsic skin aging by modulating ECM integrity.

Although basal keratinocytes are the major source of TNC in the early phase of wound healing, cultured dermal fibroblasts express more TNC than cultured keratinocytes [26,28]. Northern blot analysis revealed that TNC is expressed in two forms in the human skin [26]. The larger TNC transcript, with 7500 nucleotides, is more abundant than the smaller TNC transcript, with 5800 nucleotides, in skin tissues and fibroblasts [26]. Two TNC polypeptides of 320- and 220-kDa were detected in dermal fibroblasts [22]. Recombinant TNC polypeptides secreted from BHK cells were detected as 320- and 220-kDa bands corresponding to the 2201- and 1564-amino acid residue isoforms, respectively [23]. We also detected two main TNC variants, the more abundant 330-kDa TNC-L and the less abundant 240-kDa TNC-S forms, in conditioned media from human dermal fibroblasts by western blotting. Based on our results that TNC-L and TNC-S forms detected in dermal fibroblasts are the same in apparent MW as recombinant TNC-2201 and TNC-1564, TNC-L and TNC-S likely correspond to TNC-2201 and TNC -1564, respectively.

Different TNC isoforms can activate different cell signaling by modulating interactions with ECM components and cell surface receptors. The larger MW isoforms of TNC are associated with the induction of cell proliferation and migration by interacting with fibronectin, annexin II, and syndecan-4 [29,30]. In contrast, the smaller MW isoforms bind to fibronectin with high affinity and promote cell adhesion through the formation of focal adhesions [31,32,33]. When we treated foreskin fibroblasts with recombinant human TNC-2201 and TNC-1564 forms, both increased expression of type I collagen and reduced expression of MMP-1 without a significant discrepancy.

Although TNC is widely expressed in the embryo, *Tnc*-deficient mice develop normally and grow without any noticeable phenotype [34,35]. Later in life, some defects such as reduced fibrosis and altered inflammatory responses have been reported in these mice [21,36,37,38]. TNC deficiency reduced inflammation, TGF-β expression, and fibrosis in a murine chronic hepatitis model [20]. TNC deficiency lowered SMAD-mediated fibrosis in a murine lung injury model [21] and bleomycin-induced skin fibrosis model [16]. Similarly, we found that TNC activates phosphorylation of SMAD2/3 in foreskin fibroblasts. Interestingly, the SMAD2 phosphorylation peaked after TNC treatment with a delay than after TGF-β1 treatment, namely 90 min and 30 min after the treatment, respectively. Delayed stimulation of SMAD2/3 by TNC compared with TGF-β was recently reported in mammary fibroblasts [15]. These results suggest that TNC and TGF-β1 activate SMAD2 and induce type I collagen expression through different mechanisms.

Integrins activate latent TGF-β and the TGF-β signaling pathway [24]. For example, integrin αvβ6 activates latent TGF-β1 by binding to the RGD motif of latency-associated peptide β1 and causes actin-dependent physical deformation of the TGF-β large latent complex, exposing TGF-β to TGF-β receptors on adjacent cells [39]. A recent study reported that TNC-induced SMAD2/3 phosphorylation in an integrin αvβ1-dependent manner in mammary fibroblasts [15]. In addition, integrins are known to induce TGF-β and collagen expression through the activation of FAK and p42/p44 MAPK in mesangial cells [40]. However, we observed that the integrin inhibitors, RGD peptide and TC-I15, did not affect TNC-induced SMAD activation in foreskin fibroblasts. Interestingly, we found that incubation with cycloheximide significantly reduced TNC-induced SMAD2 phosphorylation, whereas it did not affect TGF-β1-induced SMAD2 phosphorylation. These results suggest that TNC-induced SMAD2 phosphorylation is caused by a newly synthesized signaling molecule. We found that TNC induced TGF-β1 mRNA expression in foreskin fibroblasts and that treatment with a TGF-β neutralizing antibody abolished TNC-induced SMAD2 activation. These results show that TNC induces type I collagen in foreskin fibroblasts at least in part by inducing TGF-β1 and activating SMAD2/3 through the TGF-β receptors.

We found that TNC suppressed MMP-1 via TGF-β1 induction in foreskin fibroblasts, whereas it induced type I collagen. Consistent with our finding, TGF-β signaling suppresses MMP-1 by binding to TGF-β inhibitory elements found in promoters of some *MMP* genes in fibroblasts [41]. However, TNC was reported to induce MMP-1 expression via MEK1 activation in chondrosarcoma cells [42] and activate ERK, JNK, and p38 MAPK, and integrin β1 and β3 in airway smooth muscle cells [43]. We assume that modulation of MMP-1 by TNC is dependent on cell types and environments.

The skin is an organ that is most strongly influenced by external and internal factors and plays an important role in maintaining the body’s homeostasis. As the skin ages, the skin becomes thinner and less elastic, and wrinkles appear. Compared to photoaging, which is strongly affected by UV and external factors, intrinsic aging is influenced by changes in gene expression and metabolic factors [44,45,46].

Altered TNC expression or function is associated with fibrosis in animal models [18]. For example, experimental acute lung injury, myocardial infarction, and liver and corneal injuries are associated with increased TNC accumulation and decreased resulting fibrosis in *Tnc* KO mice [20,21,47]. We also noted a decrease in dermal thickness and collagen intensity in skin tissues from young *Tnc* KO mice compared with young WT mice, although the decrease was not statistically significant. Such a small difference can be caused by a compensatory mechanism in young *Tnc* KO mice. In fact, the expression of tenascin W, which can complement with TNC, was increased in dermal fibroblasts from *Tnc* KO mice [48]. To overcome this limitation, we demonstrated that TNC increased type I collagen expression using the 3D culture of fibroblasts. These results provide compelling evidence that TNC strengthens ECM integrity in 3D matrix environments such as the skin.

## 4. Materials and Methods

### 4.1. Reagents and Antibodies

Recombinant human TGF-β1 was purchased from PeproTech (Princeton, NJ, USA). SB431542 was purchased from Selleckchem (Houston, TX, USA). RGD peptide was purchased from Santa Cruz Biotechnology (Dallas, TX, USA). TGF-β neutralizing antibody was purchased from R&D Systems (Minneapolis, MN, USA). Anti-TNC antibodies for IHC staining and western blot analysis were purchased from Santa Cruz Biotechnology and Abcam (Cambridge, UK), respectively. Anti-GAPDH antibody was from AbClone (Seoul, Korea). Anti-SMAD2 (SMAD family member 2), anti-phospho-SMAD2, anti-SMAD3, and anti-phospho-SMAD3 antibodies were from Cell Signaling Technology (Danvers, MA, USA). Anti-pro-COL1A1 and anti-MMP-1 antibodies have been described previously [49]. Horseradish peroxidase (HRP)-conjugated goat anti-mouse IgG, goat anti-rabbit IgG, and rabbit anti-goat IgG antibodies were purchased from KOMA Biotech (Seoul, Korea). Rhodamine Red-X-conjugated goat anti-mouse IgG and Alexa Fluor 488-conjugated rabbit anti-goat IgG antibodies were purchased from Invitrogen (Carlsbad, CA, USA).

### 4.2. Cell Culture

Foreskin fibroblasts (Welskin, Seoul, Korea) and dermal fibroblasts [50] were grown in Dulbecco’s modified Eagle’s medium (DMEM; Gibco, Waltham, MA, USA) supplemented with 10% fetal bovine serum (FBS; Gibco). Monkey kidney-derived COS-1 cells were grown in DMEM supplemented with 5% FBS. All the cells were grown at 37 °C under 5% CO_2_ and 95% air.

### 4.3. Acquisition of Mouse and Human Skin Tissues

Dorsal skin tissues were obtained from young (3 months old) and old (24 months old) female albino hairless (Skh-1) mice (Orient Bio, Seoul, Korea). Punch biopsy specimens (8 mm) were obtained from the buttock skin of young (aged 32, 34, and 35 years) and older (aged 72, 74, and 77 years) females without current or prior skin diseases. The skin tissues were fixed in 10% formalin for histological analysis, frozen in liquid nitrogen, and stored at −70 °C for RNA analysis. All procedures involving mice and human subjects received prior approval from the Institutional Animal Care and Use Committee and the Institutional Review Board, respectively. All the human subjects provided written informed consent.

### 4.4. RNA Isolation and RT-PCR Analysis

Total RNA isolation, cDNA synthesis, and RT-PCR analysis were performed as previously described with minor modifications [51,52]. Total RNA was isolated from the mouse skin tissues and fibroblasts using TRIzol (Invitrogen). cDNAs were synthesized from 1 µg of total RNA using oligo (dT)_15_ primer and AMV RT system (Promega, Madison, WI, USA) according to the manufacturer’s instructions. The amplification reaction was performed in a final volume of 10 µL, which consisted of 1 pM of each of the 5′ and 3′ primers, 0.2 mM dNTPs, 1 × PCR buffer, 50 unit/mL Taq polymerase, and cDNAs synthesized from 0.1 µg total RNA. PCR consisted of 21 to 30 cycles of the following steps: denaturation at 94 °C for 30 s, annealing at the appropriate annealing temperature (Supplementary Table S1) for 60 s, and extension at 72 °C for 20 s. The PCR products were detected by electrophoresis on a 5% polyacrylamide gel and visualized by staining with ethidium bromide. The expression of target genes was normalized to that of *GAPDH* gene. Real-time PCR was performed at annealing temperatures described above, using a QuantiTect SYBR Green PCR kit (Qiagen, Hilden, Germany) and the QuantStudio 3 Real-Time PCR system (Applied Biosystems, Foster City, CA, USA).

### 4.5. Histological Analysis

Skin tissues fixed in 10% formalin were embedded in paraffin wax. The paraffin-embedded samples were sectioned into 4-µm sections and mounted on silane-coated slides (Dako, Glostrup, Denmark). The mounted skin specimens were dewaxed in xylene substitutes, rehydrated with graded ethanol, and washed with distilled water. For IHC staining, the deparaffinized tissue sections were incubated with goat anti-TNC antibody (1:200) in a humidified chamber at 4 °C for 16 h. After washing with phosphate-buffered saline (PBS), the sections were visualized using an LSAB kit (Dako), which incorporated a biotinylated secondary antibody and horseradish peroxide-streptavidine conjugate. 3-amino-9-ethylcarbazole was used as the chromogenic substrate, and the sections were counterstained with Mayer’s hematoxylin. Control staining was performed with normal goat IgG antibody and showed no immunoreactivity (data not shown). For IF staining, the deparaffinized tissue sections were stained with goat anti-TNC antibody and Alexa Fluor 488-conjugated rabbit anti-goat IgG antibody for 1 h at 25 °C. Nuclei were counterstained with 4′,6-diamidino-2-phenylindole. For hematoxylin and eosin staining, deparaffinized tissue sections were incubated with absolute alcohol and washed with distilled water. Each tissue section was stained with Harris hematoxylin solution and differentiated in 1% acid alcohol. After bluing with 0.2% ammonia water for 1 min, the sections were counterstained with eosin-phloxine solution and dehydrated with 95% alcohol. Finally, the tissue sections were mounted with a xylene-based mounting medium.

### 4.6. Western Blot Analysis

Conditioned media were collected from cultured cells and the cell debris was removed by centrifugation at 2000× *g* for 3 min. Cells were washed with PBS twice and lysed with 1× sodium dodecyl sulfate (SDS) sample buffer for analysis of GAPDH or with radioimmunoprecipitation assay buffer (50 mM Tris-HCl, pH 7.4, 150 mM NaCl, 1% NP−40, 0.5% sodium deoxycholate, and 0.1% SDS) containing 1 mM NaF, 1 mM NA_3_VO_4_, and SIGMAFAST^TM^ protease inhibitor tablet (Sigma-Aldrich, St. Louis, MO, USA) for analysis of signaling proteins. Protein samples were resuspended in 1× SDS sample buffer (50 mM Tris-HCl, pH 6.8, 2% SDS, 0.1% bromophenol blue, and 10% glycerol) containing 100 mM β-mercaptoethanol, boiled for 2 min, and then resolved by SDS-PAGE. Proteins in the gel were blotted onto a polyvinylidene difluoride membrane (Millipore, Billerica, MA, USA). The blot was incubated with primary and secondary antibodies. Immunoreactive signals were detected with Immobilon Western Chemiluminescent HRP Substrate (Millipore) and LAS-3000 (Fujifilm, Tokyo, Japan).

### 4.7. Construction of TNC Expression Vectors

To generate pcDNA3.1-TNC-1564-His encoding full-length human TNC-1564 isoform (GenBank XM_005251975) with a C-terminal His tag, a 4730-bp DNA fragment of pBS-HxB.S [23], including TNC-1564 cDNA was PCR-amplified using PrimeSTAR GXL DNA polymerase (TaKaRa, Shiga-ken, Japan), and a primer pair: 5′-GAC*GCTAGC*CACC**ATG**GGGGCCATGACTCA-3′, which includes an NheI site (italicized) and nucleotides 309-329 of GenBank XM_005251975 containing a start codon (bold), and 5′-AT*GGTACC***TTA**ATGATGATGATGATGATGTGCCCGTTTGCGCCTGCCT-3′, which includes a KpnI site (italicized), a stop codon (bold), His tag (underlined), and nucleotides 5004-4986 of Genbank XM_005251975. The PCR product was cleaved with NheI and KpnI and then ligated into the NheI and KpnI sites of pcDNA3.1 plasmid.

To generate pcDNA3.1-TNC-2201-His encoding full-length human TNC-2201 isoform (GenBank NM_002160) with a C-terminal His tag, a 3355-bp fragment corresponding to nucleotides 2706-6060 of GenBank NM_002160 sequence was isolated by cleavage of pBS-HxB.L [23] including TNC-2201 cDNA, with BstEII. Subsequently, the 3355-bp BstEII fragment was ligated into the 8686-bp BstEII fragment of pcDNA3.1-TNC-1564-His lacking a 1444-bp BstEII fragment corresponding to nucleotides 2706-4149 of GenBank XM_005251975 sequence. All the generated constructs were sequenced to confirm that their sequences were error-free.

### 4.8. Purification of Recombinant Human TNC

Subconfluent COS-1 cells were transfected with pcDNA3.1-TNC-2201-His or pcDNA3.1-TNC-1564-His using the calcium phosphate method for 16 h, subsequently the cells were incubated with DMEM containing 5% FBS for 24 h. The cells were then washed with PBS three times and incubated with serum-free DMEM for 48 h. Recombinant human TNC-2201 or TNC-1564 with C-terminal His tags were purified from the serum-free conditioned medium using Ni^2+^-NTA column chromatography (QIAGEN), following the manufacturer’s recommendations. The eluted recombinant TNC-2201 or TNC-1564 polypeptides were dialyzed against PBS and stored as frozen aliquots at −80 °C.

### 4.9. Construction of pGEM-TGF-β1, 2, and 3 Vectors

To generate pGEM-TGF-β1, 2, and 3 vectors for use as positive controls in RT-PCR, 161-bp *TGF-β1*, 162-bp *TGF-β2*, and 134-bp *TGF-β3* cDNA fragments were PCR-amplified using the primer pairs for RT-PCR (Supplementary Table S1, Taq DNA polymerase, and foreskin fibroblast cDNA as template. The cDNA fragments were then ligated into pGEM-T vector (Promega).

### 4.10. Treatment of Fibroblasts with TNC or TGF-β1 in the Presence or Absence of Signaling Blockers

To analyze the expression levels of target genes, subconfluent foreskin fibroblasts were incubated in media containing 10% FBS for 12 h. The medium was replaced with fresh serum-free media containing 0.1% bovine serum albumin (BSA) with purified TNC (2 µg/mL) or TGF-β1 (3 ng/mL), and the cells were incubated for 12 h for mRNA analysis or for 24 h for protein analysis.

To analyze the phosphorylation of the signaling proteins, subconfluent foreskin fibroblasts were cultured in serum-free media for 10 h. The medium was replaced with fresh serum-free media containing 0.1% BSA. After 2 h, the cells were further incubated with either TNC (2 µg/mL) or TGF-β1 (3 ng/mL) for 90 min unless specified. In experiments involving signaling blockers, 10 µM SB431542, 100 µg/mL RGD peptide, 2 µM TC-I15, 10 µg/mL cycloheximide, or 3 µg/mL TGF-β neutralizing antibody, were added 10 min prior to the treatment of the cells with TNC or TGF-β1.

### 4.11. Synthesis of Type I Collagen in Fibroblasts Grown in 3D Culture

3D culture and immunofluorescence staining of fibroblasts were performed as previously described with minor modifications [53]. For 3D culture of fibroblasts, foreskin fibroblasts (8 × 10^5^ cells/mL) were trypsinized and resuspended in 2.8 mg/mL collagen I solution (4 mg/mL rat tail Corning^®^ collagen I:10× DMEM:10× reconstitution buffer [260 mM NaHCO_3_, 200 mM HEPES, and 50 mM NaOH]:2 µg/mL TNC = 7:1:1:1). The mixture (0.15 mL) was loaded onto a glass-bottom (35 mm × 10 mm, hole 13 Φ) dish (SPL Life Sciences, Pocheon, Korea). After allowing the solution to gel for 1 h at 37 °C, 2 mL of phenol red-free DMEM (Hyclone, South Logan, UT, USA) was added, and the collagen-embedded cells were incubated for 24 h at 37 °C with 5% CO_2_ and 95% air. For nuclear staining, the cells were incubated with Hoechst 33258 (2 μg/mL) for 30 min. After incubation, the cells were fixed in 3.7% paraformaldehyde for 30 min, permeabilized with 0.2% Triton-X 100 for 30 min, blocked with 3% BSA for 30 min, and immunostained with mouse anti-pro-COL1A1 antibody (1:50) overnight at 4 °C. Samples were washed with PBS and incubated with Rhodamine Red-X-conjugated goat anti-mouse IgG (H+L) antibody (1 unit/mL). Images were obtained using a confocal microscope (LSM880; Zeiss, Oberkochen, Germany) with a 20× objective lens and Zen software version 2.3 (Zeiss).

### 4.12. Statistical Analysis

All data are expressed as the mean ± standard deviation (SD) of at least three independent experiments. Statistical significance was analyzed by Student’s *t*-test, and *p* < 0.05 was considered statistically significant.

## 5. Conclusions

In this study, we have shown that TNC is downregulated in aged skin tissues of mice and humans compared to young tissues. Expression of two major TNC isoforms, TNC-L and TNC-S, was observed in primary dermal fibroblasts. Recombinant TNC polypeptides, corresponding to TNC-L and TNC-S, increased the expression of type I collagen and decreased the expression of MMP-1, without discrepancy. A recombinant TNC polypeptide, corresponding to TNC-L, induced phosphorylation of SMAD2 and SMAD3 in fibroblasts. In addition, TNC induced transcription of *TGF-β1* mRNA and activated the TGF-β signaling pathway, whereby the expression of type I collagen in fibroblasts was upregulated. Moreover, TNC promoted biosynthesis of type I collagen in the 3D culture system. Based on our findings, we propose that TNC expression in the skin plays a complementary role in the loss of ECM integrity that occurs in the intrinsic aging process.

## Supplementary Materials

Supplementary Materials can be found at https://www.mdpi.com/1422-0067/21/22/8693/s1. There were one Table S1 containing primer sequences used for RT-PCR, and one Figure S1 containing results of dermal thickness and collagen intensity in skin tissues of *Tnc* knockout and wild-type mice in Supplementary Materials.

## Abbreviations

3D	Three-dimensional
DAPI	4′,6-diamidino-2-phenylindole
DMEM	Dulbecco’s modified Eagle’s medium
ECM	Extracellular matrix
EGF	Epidermal growth factor
MMP	Matrix metalloproteinase
IF	Immunofluorescence
IHC	Immunohistochemistry
KO	Knockout
R-SMAD	Receptor-regulated SMAD
RT-PCR	Reverse transcription-polymerase chain reaction
TGF-β	Transforming growth factor-β
TNC	Tenascin C
WT	Wild-type
