# Supplementary material for: Effects of Tenascin C on the Integrity of Extracellular Matrix and Skin Aging
Source: Int J Mol Sci. 2020 Nov 18;21(22):8693. doi: 10.3390/ijms21228693 (PMC7698786; doi:10.3390/ijms21228693)
Supplement: Supplementary file 1 [file ijms-21-08693-s001.pdf]

International Journal of
Molecular Sciences

MDPI

Supplementary Materials

Effects of tenascin C on the integrity of extracellular matrix and skin aging

Young Eun Choi [1], Min Ji Song [2], Mari Hara [3], Kyoko Imanaka-Yoshida [3,4], Dong Hun Lee [2,5], Jin Ho Chung [2,5], and Seung-Taek Lee [1,*]

[1] Department of Biochemistry, College of Life Science and Biotechnology, Yonsei University, Seoul 03722, Republic of Korea; choii03271452@gmail.com
[2] Department of Dermatology, Seoul National University College of Medicine, Seoul 03080, Republic of Korea; minjisong@snu.ac.kr (M.J.S.); ivymed27@snu.ac.kr (D.H.L.); jhchung@snu.ac.kr (J.H.C.)
[3] Department of pathology and Matrix Biology, Mie University Graduate School of Medicine, Tsu 514-8507, Japan; marihara@doc.medic.mie-u.ac.jp (M.H.); imanaka@doc.medic.mie-u.ac.jp (K.I.-Y.)
[4] Mie University Research Center for Matrix Biology, Tsu 514-8507, Japan
[5] Institute of Human-Environment Interface Biology, Medical Research Center, Seoul National University, Seoul 03080, Republic of Korea
* Correspondence: stlee@yonsei.ac.kr; Tel.: +82-2-2123-2703

Supplementary Table S1. Primer sequences used for reverse transcription-polymerase chain reaction of mouse *Tnc* and human *COL1A1, COL1A2, MMP1, TGF-β1, TGF-β2, TGF-β3, and GAPDH* mRNAs.

Gene Name	Nucleotide Sequence	Nucleotide Position	Annealing Temp. (℃)	GenBank #
m*Tnc*	5'-ACTGTCATCGTGTCAACCTGATGG-3'<br>5'-TCCAGATTTCGGAAGTTGCTGG-3'	6289-6312<br>6430-6409	52.5	NM_011607
h*COL1A1*	5'-ACAGCGTCACTGTCGATGGCTG-3'<br>5'-GGAGGGAGTTTACAGGAAGCAGACAG-3'	4341-4372<br>4522-4497	55	NM_000088
h*COL1A2*	5'- GAGGGCAACAGCAGGTTCACTTACAC-3'<br>5′-GTCAGCACCACCGATGTCCAA AG-3'	4044-4069<br>4196-4174	55	NM_000089
h*MMP1*	5'-GTACTGATATAATTTAGTTC -3'<br>5'-GTTATCCCTTGCCTATCTAG -3'	1656-1675<br>1908-1889	45	NM_002421
h*TGF-β1*	5′-ACACGCAGTACAGCAAGGTCCTGG- 3′<br>5-GGAGCGCACGATCATGTTGGAC-3′	1876-1899<br>2036-2015	67	NM_000660
h*TGF-β2*	5′-GCAGAAGAGGTATCCTCATGCTGGG-3′<br>5′-CAAACAACTTCACTTTGGATTCCCG- 3′	4481-4505<br>4642-4618	67	NM_003238
h*TGF-β3*	5′-TACAACACTCTGAACCCTGAAGCATC-3′<br>5′-CAAGACTTCACCACCATGTTGGAG-3′	2195-2220<br>2328-2305	62	NM_003239
h*GAPDH*	5'-ACTGCTTAGCACCCCTGGCCA-3'<br>5'-TTGGCAGTGGGGACACGGAAG-3'	488-508<br>740-720	55	BC023632

h: human, and m: mouse

Supplementary Figure S1

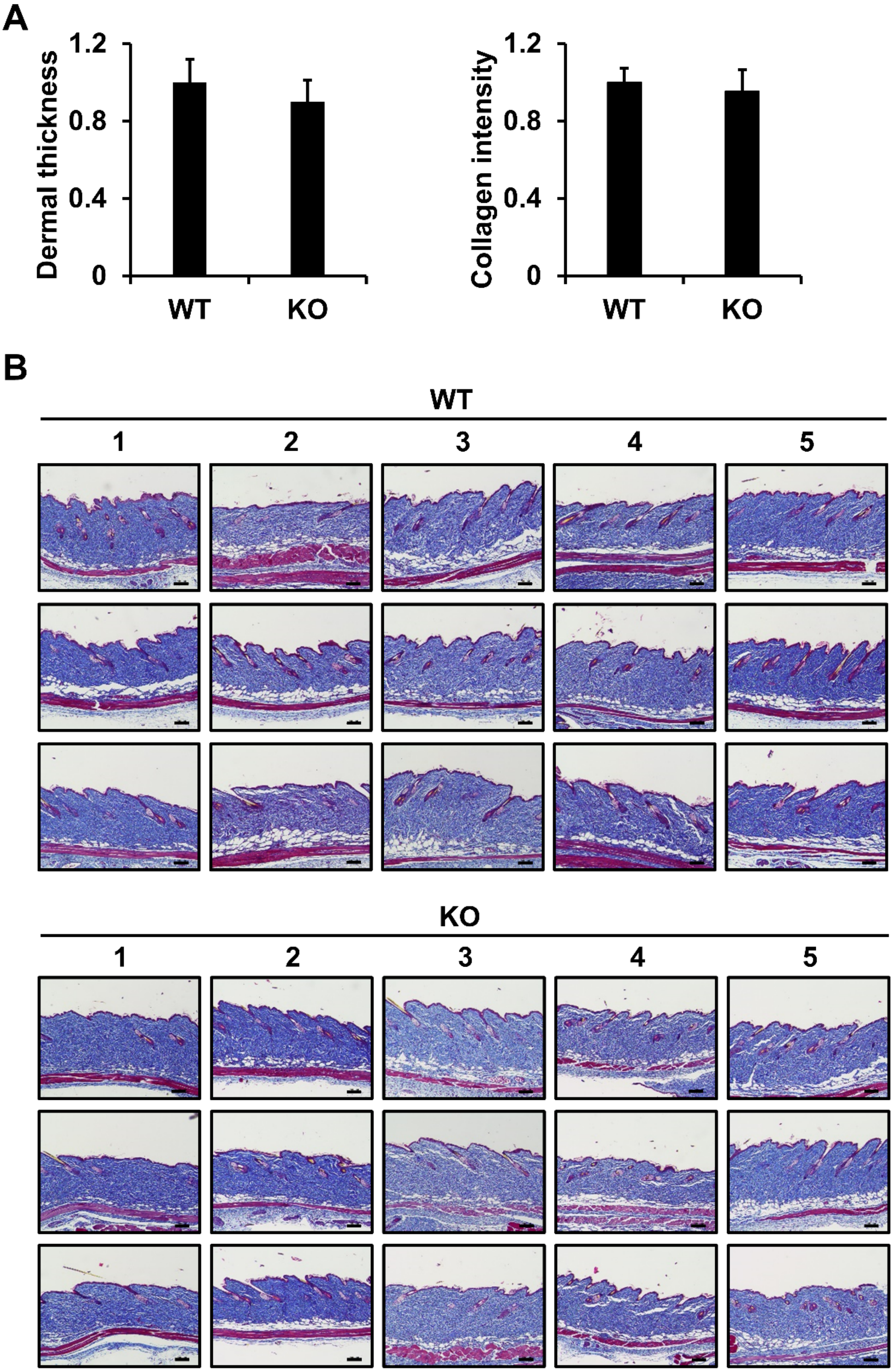

Supplementary Figure 1. Analysis of dermal thickness and collagen intensity in skin tissues of *Tnc* knockout and wild-type mice. Formalin-fixed and paraffin-embedded sections of skin tissues from *Tnc* knockout (KO) and wild-type (WT) C57BL6 mice (6 weeks old) were deparaffinized and stained with Masson's trichrome [1]. Dermal thickness [2] and collagen intensity [3] in the dermis were measured as the width or staining intensity at five locations in each tissue section using Image J software (National Institutes of Health, Bethesda, MD, USA). Five mice from each group and three separate blocks of skin tissues from each mouse were evaluated. (A) Graphs showing relative dermal thickness and collagen intensity in five *Tnc* KO mice compared with WT mice. (B) Three blocks of skin tissues from five *Tnc* KO or WT mice, each stained with Masson's trichrome are shown. Bar = 100 μm.

Supplementary References

1. Kim, J. E.; Song, D.; Kim, J.; Choi, J.; Kim, J. R.; Yoon, H. S.; Bae, J. S.; Han, M.; Lee, S.; Hong, J. S.; et al. Oral Supplementation with Cocoa Extract Reduces UVB-Induced Wrinkles in Hairless Mouse Skin. *J. Invest. Dermatol.* **2016,** *136*, 1012-1021.
2. Terao, M.; Tani, M.; Itoi, S.; Yoshimura, T.; Hamasaki, T.; Murota, H.; Katayama, I. 11beta-hydroxysteroid dehydrogenase 1 specific inhibitor increased dermal collagen content and promotes fibroblast proliferation. *PLoS One* **2014,** *9*, e93051.
3. Chen, Y.; Yu, Q.; Xu, C. B. A convenient method for quantifying collagen fibers in atherosclerotic lesions by ImageJ software. *Int. J. Clin. Exp. Med.* **2017,** *10*, 14904-14910.
